# Diabetic Cardiomyopathy: Current Approach and Potential Diagnostic and Therapeutic Targets

**DOI:** 10.1155/2017/1310265

**Published:** 2017-03-21

**Authors:** Georgiana-Emmanuela Gilca, Gabriela Stefanescu, Oana Badulescu, Daniela-Maria Tanase, Iris Bararu, Manuela Ciocoiu

**Affiliations:** ^1^Department of Pathophysiology, Faculty of Medicine, University of Medicine and Pharmacy “Grigore T. Popa” Iasi, Iasi, Romania; ^2^Gastroenterology Department, “Sf. Spiridon” County Clinical Emergency Hospital, University of Medicine and Pharmacy “Grigore T. Popa” Iasi, Iasi, Romania; ^3^3rd Internal Medicine Clinic, “Sf. Spiridon” County Clinical Emergency Hospital, University of Medicine and Pharmacy “Grigore T. Popa” Iasi, Iasi, Romania

## Abstract

Although ischemic heart disease is the major cause of death in diabetic patients, diabetic cardiomyopathy (DCM) is increasingly recognized as a clinically relevant entity. Considering that it comprises a variety of mechanisms and effects on cardiac function, increasing the risk of heart failure and worsening the prognosis of this patient category, DCM represents an important complication of diabetes mellitus, with a silent development in its earlier stages, involving intricate pathophysiological mechanisms, including oxidative stress, defective calcium handling, altered mitochondrial function, remodeling of the extracellular matrix, and consequent deficient cardiomyocyte contractility. While DCM is common in diabetic asymptomatic patients, it is frequently underdiagnosed, due to few diagnostic possibilities in its early stages. Moreover, since a strategy for prevention and treatment in order to improve the prognosis of DCM has not been established, it is important to identify clear pathophysiological landmarks, to pinpoint the available diagnostic possibilities and to spot potential therapeutic targets.

## 1. Introduction 

Cardiovascular disease represents the leading cause of death and disability among diabetic patients [1].

The impact of diabetes on cardiac function is slow and silent, currently diagnosed only when there is a certain degree of dysfunction. Thus, the medical management and lifestyle interventions must take into account the potential impairment of left ventricular function in a patient with diabetes, even without underlying arterial hypertension, valvular or congenital cardiomyopathy, or coronary artery disease, a condition which is now framed as a distinct entity, namely, diabetic cardiomyopathy (DCM) [2]. It is important to be aware that diabetic cardiac disease may result from both type 1 and type 2 diabetes, consequent to various structural changes, eventually leading to heart failure if left undiagnosed and untreated. As a consequence, early detection and progression prevention of diabetic cardiomyopathy are essential for this patient category, especially considering the worse prognosis of heart failure among diabetic patients [3].

Evidence that DM represents a stronger predictor of mortality than coronary artery disease (CAD) in cohorts with heart failure [4] suggests that diabetic hearts have accentuated cellular damage and severely reduced cellular reserve and are more exposed to future cardiac events leading to decompensation and failure [5].

DCM comprises several morphological and structural myocardial changes, which are induced through activation of various changes, with mechanical dysfunction as a fundamental change, consequent to unbalance between oxidants versus antioxidants, in favor of a prooxidative stress [6]. Moreover, metabolic and functional alterations lead to a silent development of DCM, consisting of augmented free fatty acid (FFA) metabolism and modified intracellular signaling in cardiomyocytes, with consequent inefficient energy production and deficient cardiomyocyte contractility. The resulting diastolic and systolic dysfunction in DCM is also due to microvascular modifications with myocardial fibrosis and steatosis and also remodeling of the extracellular matrix [7].

## 2. Current Diagnostic Landmarks in DCM

DCM is described as typical heart failure with preserved ejection fraction (EF), considering diastolic dysfunction as the first hallmark of DCM, together with concentric cardiac hypertrophy. Consequently, there has been also proposal of a four-stage classification of DCM, including clinical and echocardiographic changes, but also cellular mechanisms are involved, offering landmarks for diagnosis in clinical practice [8]. Diastolic dysfunction has been considered the first identifiable functional change in DCM; although impaired relaxation might be influenced by several factors (such as age and BMI), some studies demonstrated that, in diabetic patients, left ventricle (LV) relaxation is impaired even in the absence of coronary artery disease (CAD) or arterial hypertension (HTA) [7].

Studies have highlighted that even when diastolic function is normal and left ventricle (LV) EF is preserved, there still exists a systolic LV strain alteration (MAPSE and longitudinal systolic LV strain), leading to the idea that diastolic dysfunction should not be considered the first sign of subclinical diabetic cardiomyopathy [9].

This finding is also supported by the use of myocardial performance index (MPI) in order to evaluate global cardiac contractility, since it was demonstrated that an altered MPI is the earliest echocardiographic change in DCM, with recorded higher values in DCM patients compared with controls [10]. Moreover, MPI might be useful in assessing the metabolic control or in indicating the necessity to early initiate pharmacologic therapy in T2DM, also offering the possibility to monitor the potential reversion of initial contractility dysfunction in DCM as response to optimal metabolic control [9].

Since evaluating asymptomatic diabetic patients, in the absence of risk factors, is challenging in everyday practice, there has been proposal of a stage-adapted model of DCM including four stages, considering pathophysiological features, echocardiographic changes, serological biomarkers, such as matrix metalloproteinases (MMPs), and tissue inhibitor of metalloproteinases (TIMPs). These stages comprise the following: stage 1 DCM (mixed hypertrophic and restrictive phenotype), stage 2 DCM (systolic dysfunction and dilatation), stage 3 DCM (systolic dysfunction to which microangiopathy and HTA have contributed), and stage 4 DCM (including dilatation, fibrosis, micro- and macroangiopathy) [8]. Moreover, the interrelation between other biochemical parameters and the evolution of DCM among normotensive diabetic patients has been studied, regarding the prediction potential upon LV geometry and consequent cardiac dysfunction, of parameters such as level of glycosylated hemoglobin, presence of microalbuminuria, and retinopathy; however, no clear biochemical profile with prediction potential for the development of diabetic cardiomyopathy in normotensive diabetic patient has been established [7, 11] (Table 1).

Assessing the cardiac function in diabetic patients can also be performed by cardiac MRI, not only highlighting myocardial fibrosis as a first sign of left ventricular dysfunction, but also considering the possibility to distinguish the cardiac steatosis. This second metabolic landmark can be spotlighted through MRI as an early change in the course of DCM, considering that there is an increased uptake of FFA, with exceeding FFA oxidation, leading to enhanced accumulation of myocardial lipids and consequent altered intracellular signaling [12]. The myocardial triglyceride content (but not perfusion reserve) is associated with LV diastolic dysfunction in type 2 diabetes mellitus (T2DM), even after adjusting several parameters which could lead to potential biases (such as duration of T2DM, blood pressure, or fasting blood glucose) [7, 13].

As for biomarkers evaluating the risk for cardiovascular events in diabetic patients, MMP3, osteopontin and N-terminal prohormone of B-type natriuretic peptide (NT-proBNP), and their combined value score are currently proposed to be introduced in evaluating diabetic patients, but first there is a need for more clear evidence that their use offers a more accurate risk stratification than the traditional risk factors [14].

## 3. Overview on Mechanisms Involved in Diabetic Heart Disease and Potential Therapeutic Targets

Various metabolic changes are comprised during long time evolving DM: the cardiac uptake of albumin-fatty acid is augmented; the storage and catabolism of endogenous triglycerides are increased, as well as the circulating lipoprotein levels, as a consequence of enhanced hepatic VLDL synthesis [14]. In DCM, calcium signaling is altered; hence the contraction-relaxation dynamic is affected, with consequent alteration of structural components and activation of various signaling pathways, including NF-kB, c-Jun N-terminal kinases, and p38 mitogen-activated protein kinases through oxidative modifications. There is also impairment in protein folding, leading to toxicity and potential initiation of cardiomyocyte apoptosis.

### 3.1. Free Fatty Acid Metabolism 

The fatty acid (FA) metabolism is altered during DM, leading to lipotoxic cardiac injury; FAs enter the cell both via passive diffusion and facilitated transport, which can be mediated by various FA transport proteins, including fatty acid binding proteins (FABP). Once entered the cell, FAs can be used as substrate by mitochondria to generate ATP, which can be temporarily stored after esterification or can activate PPAR, a class of nuclear mediators promoting transcription of genes coding proteins involved in FAs utilization. Overall, the myocardial FA build-up leads to decreased myocardial energy production, reduced myocyte contractility, and lipoapoptosis [15]. Among FABP, FABP4 and FABP5 are members of the intracellular lipid-binding protein family predominantly expressed in adipose tissue with evidence from experimental studies [16–18] and also in clinical studies as being involved in insulin resistance, with registering additive effect of FABP4 and FABP5 on metabolic-inflammatory CVD risk and atherosclerosis in humans [19], which might raise the potential usefulness of evaluating FABP4 and FABP5 as biomarkers of increased metabolic cardiovascular risk in diabetic patients.

Moreover, in order to investigate the FA dismetabolism and interaction with various mediators of the immune system and consequences on evolution of DCM, the role of caloric restriction was studied, both to highlight the cellular pathway and to reveal its potential therapeutic use. Toll-like Receptors (TLRs) are a component of the innate immune system, being responsible for pattern recognition and responding not only to inter- and intracellular molecules typically associated with pathogens (usually activated by bacterial lipopolysaccharide), but also to several endogenous ligands and involving adipocytes activity, with consequent metabolic interaction with myocardial cells function, since cardiomyocytes express TLR4 [20]. A ligand of TLR4 is FAs, the binding being mediated by Fetuin A (Fet A), through which lipids induce insulin resistance [21]. Fet A is a circulating negative acute-phase glycoprotein synthesized in adult human liver tissue. An association exists between high levels of Fet A and cardiovascular disease in diabetic patients and is manifested as a higher risk of cardiovascular disease in these individuals [22]. Since high levels of Fet A are correlated with higher levels of TLR2 expression at adipocyte level, this means that high Fet A level is related to inflammation during metabolic syndrome as manifested by TLRs activation [23]. It has been proved on experimental models that chronic FAs release stimulated TLR4 on adipocytes, leading to increased production of inflammatory adipokines, including interleukin-6 (IL-6), tumor necrosis factor-a (TNF-a), and monocyte chemoattractant protein-1 (MCP-1), consequently leading to increased monocyte accumulation, giving rise to impaired insulin sensitivity [24, 25]. Cohen et al. demonstrated that TLR2/4, FFAs, and Fet A are increased during diabetic cardiomyopathy, but there is possibility to achieve a significant improvement of these biomarkers through a short period of caloric restriction, with a consequent therapeutic potential [24].

The metabolic component is the central part of DCM, with high reliance on FFA and their increased beta oxidation, leading to insulin resistance and to a dysfunction of the Ca^2+^ transporter protein; intracellular Ca^2+^ level is adjusted by opening the K-ATP channel, determining a decrease in Ca^2+^ influx, which contributes to worsening cardiomyocyte contractility. These metabolic events generate intramyocardial lipotoxicity, with increased proinflammatory cytokines and consequent cardiomyocyte hypertrophy. The increased intracellular FFAs inhibit key enzymes such as pyruvate dehydrogenase, determining accumulation of glycolytic intermediates and ceramide, with its production being facilitated by TNF-*α* and potentially contributing to apoptosis. Oxidative stress is also assumed to play a role in acceleration of apoptosis, through glycosylation and phosphorylation of p53 [8, 26].

### 3.2. Cardiac Ubiquitin Proteasome System (UPS)

Regarding cellular mechanisms, a pathogenic role has been recently attributed to the cardiac ubiquitin proteasome system (UPS), responsible for the maintenance of protein homeostasis by degrading the damaged proteins such as terminally misfolded proteins and oxidized proteins. In vitro and in vivo studies show that UPS dysfunction is an early event in diabetes and it triggers pathological remodeling in diabetic hearts [27–29].

Studies on in vivo UPS functional reporter colligated with other biochemical analyses reveal that increasing activity of the cardiac UPS by overexpression of PA28*α* reduced the proteotoxic stress at cardiac level, reducing cardiac dysfunction. Consequently, proteasome dysfunction might represent a novel mechanism involved in DCM; however, the effects of diabetes on the overall cardiac UPS function and its pathophysiological role in diabetic cardiomyopathy are still to be further investigated [29].

Proteotoxic stress, through excessive production of misfolded proteins or through inhibition of proteasome or through autophagy inhibition, leads to contractility dysfunction in mouse models. It has been proved on a high fat diet-induced obesity mouse model that adiponectin deficiency leads to reduction of autophagy and worsening of cardiac dysfunction [30]. Moreover, the reduction of proteotoxic stress by improving autophagic function has been proved to have positive impact on cardiac contractility [31]. These findings altogether suggest that reducing proteotoxic stress is a key factor in preserving cardiomyocyte function and that reducing cardiac proteotoxicity might represent a therapeutic approach in DCM [29].

### 3.3. Deficiency in Calcium Handling

In the development of cardiac contractility dysfunction, deficient calcium handling plays a key role, since it controls the excitation-contraction coupling. During contraction, calcium from the sarcoplasmic reticulum represents the major source of calcium, released from ryanodine receptor; the increased calcium cytoplasmic concentration is triggering myofilament contraction. Abnormal function of RyR has been proved to contribute to various situations in which cardiac dysfunction is involved, such as cardiac hypertrophy and ischemia-reperfusion lesions, but also in streptozotocin-induced type 1 diabetes, although the pathological significance is not well characterized [32]. The reduced diastolic intracellular concentration may be due to AGE/RAGE interaction, potentially through influencing RyR activity, since RyR due to its structure rich in free thiol groups is highly susceptible to oxidative stress [33]. Yan et al. have demonstrated that AGE/RAGE induced hyperactive RyR in cardiomyocytes because of RyR's high susceptibility to oxidative stress and also that the hyperactive RyR-mediated SR leak reduced SR Ca 2+ content, resulting in a decrease in the systolic Ca 2+ transient. Moreover, a reduced mitochondrial Ca uptake may significantly decrease ATP synthesis rate, leading to reduced contractility ability and consequent DCM [34].

ROS production to which RyR function is sensitive may also be linked not only to AGE/RAGE interaction, but also to sympathetic hyperactivation coupled with hyperglycemia in T2D, leading to impaired intracellular calcium handling and myocyte contractile dysfunction [35].

## 4. Current Diagnosis of DCM

In the current clinical practice, the approach to diagnose DCM includes detecting structural and functional changes in the LV and adding proofs to exclude other cardiac disease as a potential etiology for the changes in a patient with diabetes. Most frequently, strain rate imaging and tissue Doppler imaging are used, offering the possibility to highlight the LV diastolic dysfunction during exercise stress, since the absence of echocardiographic changes at rest cannot exclude early DCM. However, there have been reports of systolic dysfunction (identified as abnormal systolic longitudinal strain rate) in the absence of diastolic dysfunction among diabetic patients [9]. In order to early identify and evaluate cardiac dysfunction in diabetic patients, MR spectroscopy represents a potential novel diagnostic tool, considering its possibility to identify myocardial metabolic changes, such as quantifying myocardial triglycerides content. Since there is increasing evidence that the myocardial metabolism is altered during T2DM, with lipotoxic injury due to lipid oversupply, it has been demonstrated that myocardial triglyceride content is independently associated with biventricular changes in myocardial systolic and diastolic functions [36].

In the early stages, there are only substructural changes in the cardiomyocytes, and the detection is possible only by very sensitive methods such as strain, strain rate, and myocardial tissue velocity [15]. As for other novel techniques with potential to highlight early changes in heart metabolism, molecular magnetic resonance imaging (MRI) with specific labeling of free radicals might be a promising tool, with current use on animal models proving increased levels of free radicals in diabetic mice compared with controls. Considering myocardial fibrosis with increased extracellular matrix deposition, with consequent interstitial fibrosis, together with augmented free radical production as landmarks in the pathophysiology of DCM, in vivo imaging technique can prove to be useful in identifying the source and also the type of free radicals generated through oxidative stress during DCM [37]. Assessment of interstitial fibrosis and steatosis by using delayed gadolinium enhancement cardiac MRI is possible but its diagnostic value has not been established.

Since normal echocardiographic findings at rest cannot exclude DCM, left ventricular diastolic dysfunction detectable by TDI (and possible also by SRI) at exercise stress may be the earliest echocardiographic sign of diabetes-induced LV dysfunction. As a consequence, there is a need to highlight earlier changes, for instance by assessing the level of interstitial fibrosis, using gadolinium-enhanced MRI. This method has not been confirmed as a valuable diagnostic tool and, consequently, the characterization of other metabolic changes in the cardiomyocyte is required; since there is evidence of reduced phosphocreatine/ATP ratio in the myocardium of diabetic patients, compared with control subjects, using Magnetic Resonance Spectroscopy with ^31^P or ^1^H for determining it might represent a valuable tool. Using 1 H-MRS, it has been demonstrated that increased myocardial triglyceride content (i.e., myocardial steatosis) was associated with LV diastolic dysfunction in diabetic patients. In other study, myocardial steatosis was independently correlated with more pronounced impairment of ventricular contractility (evaluated by two-dimensional speckle tracking imaging) in patients with uncomplicated diabetes mellitus [36, 38–40].

## 5. Future Directions for Diagnosis and Potential Treatment Targets for DCM

### 5.1. miRNA

 The potential role of miRNAs in various pathologies has been recently emphasized, as a new perspective for diagnosis and treatment target has received considerable attention in biomedical research as a possible alternative for treatment of disease.

Changes in the plasma level of cardiac biomarkers may reflect several myocardial metabolic changes. Although micro-Ribonucleic acids (miRNAs) are small noncoding RNA molecules, they play a key role in modulating gene expression and their altered level was found in the cardiomyocytes of experimental diabetes models. The expressions of several miRNAs are altered by matrix metalloproteinases (MMPs), which also play an important role in degrading extracellular matrix; the increased turnover of extracellular matrix proteins and alteration of MMPs levels are strongly correlated with active cardiac remodeling; furthermore, elevated level of MMP9 and reduced level of TIMPs are associated with myocardial fibrosis.

Among this group of noncoding RNA, miR-223 is associated with regulation of glucose transporter 4 (GLUT4) expression in cardiomyocytes. Insulin resistance promotes downregulation of GLUT4 at the plasma membrane level and upregulation of miR-223. miR-223 might also have a role in increasing nuclear factor IA expression but without affecting phosphoinositide 3-kinase signaling and AMP kinase activity. Considering that miRNAs act as stress response genes and are necessary for maintaining the strength of physiologic responses (to restore GLUT4 expression and normal glucose uptake) when pathophysiologic condition arises (in this case the insulin resistance) and that specifically miR-223 has the ability to upregulate target genes such as GLUT4 in adult cardiomyocytes, they might represent a valuable therapeutic target [26].

miRNAs as highly conserved, small noncoding RNAs are evaluated as a potential regulator of more than 200 different transcripts, with a precisely controlled expression, depending on tissue-specific conditions. Specifically, for miR-1, miR-499, miR-133a, and miR-133b several intracellular targets have been identified, related to imbalance of oxidant injury versus antioxidant defense. Better knowledge on tissue-specific roles of individual miRNAs under hyperglycemic conditions might reveal new diagnostic and early therapeutic possibilities for T2DM complications. Data from experimental studies emphasize the contribution of miR-21 in stimulating MAP kinase signaling in mouse fibroblasts, thus promoting fibrosis and contractility alterations as features of DCM in diabetic animal models [6, 15, 41].

miRNA is reported to be involved in regulating transcription of various kinases, including extracellular signal-regulated kinases (ERK) in diabetic conditions and thus in modulating ERK1/2 derived-pathway which opposes oxidative stress-induced insulin resistance in cardiomyocytes [42]. This is of particular interest in preventing DCM, since ROS are reported to activate ERKs through mechanisms dependent also on magnitude and compartmentalization of ERK MAPK activity that still require further research [43].

### 5.2. Exosomes

Regarding DCM and the importance of miRNAs in this disease, exosomes emerge as potential biomarkers; although there is much to be elucidated regarding the role of these nanovesicles in DCM, it has been suggested that not only they represent the main vehicle for the plasmatic miRNAs, but also miRNAs are bound to complexes such as high density lipoproteins [44, 45].

Recently, it has been suggested that there is a strong relationship between nutrient sensing and exosome release. Under glucose deprivation conditions, there was an increased exosomes production from cardiomyocytes, with these nanovesicles containing glucose transporters and enzymes involved in glycolysis, consequently leading to an increase in glucose uptake and use with resulting pyruvate production in endothelial cells. Thus, cardiomyocyte-derived exosomes can regulate glucose transport in endothelial cells, the vesicle mechanism involving GTPases (guanosine triphosphatases) and several Rab (Ras-related proteins in brain) GTPases. This complex process comprises a convergence of pathways, integrating autophagy, vesicle transport, and exosomal synthesis and secretion [46].

Nutrient sensing and exosome release are closely related processes, considering the significant overlap in the molecular machinery used in exocytosis and exosome trafficking. The absence of glucose generates stress signals, leading to a convergence of the GLUT trafficking pathway and the exosome secretion pathway. Outlining several interconnected molecular mechanisms involved in multivesicular body biology, it is also proposed that glucose uptake from the endothelium to cardiomyocytes could be regulated, at least in part, by short range exosome communication. As a consequence, exosomes represent potential biomarkers and they might also represent therapeutic targets or agents that could reverse the impaired insulin signaling observed in DCM [45].

### 5.3. Mimetic Peptides Targeting Calcium Channels

There is intense experimental research on potential therapeutic approaches of DCM, and considering the essential role of intracellular calcium imbalance in contractile dysfunction, mimetic peptides (MP) targeting L-type calcium channels (LTCCs) have been tested. Therefore it has been proved on experimental model that the LTCC Ca_v_*β*2 chaperone regulates Ca^2+^ channel density at plasma membrane level, therefore correcting LTCC life cycle alterations and promoting proper contractility. The conformational change in Ca_v_*β*2 is Akt phosphorylation-dependent; consequently to calcium influx, a complex intracellular pathway is generated, including the reduction of Ca_v_*α*1.2 retrograde trafficking and protein degradation through prevention of dynamin-mediated LTCC endocytosis, promoting Ca_v_*β*2 anterograde trafficking and further Ca_v_*α*2 transcription. This type of proposed MP is a positive Ca^2+^ modulator, which also means that it increases arrhythmogenesis risk. Therefore, further studies addressing pharmacokinetic analysis are required; since supraphysiological levels of Ca_v_*α*1.2 and Ca_v_*β*2 exert detrimental effects regarding myocyte function, effects which are beyond the linear correlation between Ca_v_*α*1.2 and Ca_v_*β*2 are highlighted by preliminary data [47]. Moreover, several tissue-specific elements should be tackled; since Ca_v_*β*2 has a broad tissue distribution, not being limited to cardiac tissue, thus possibly generating several issues, difficulty which might be overcome by using functionalized nanoparticles [47, 48] allow specific cardiac delivery.

## 6. Conclusions

It is important to achieve an early diagnosis of DCM in asymptomatic diabetic patients in order to prevent the development of irreversible morphological changes, such as fibrosis, leading to impaired contractility. As a current practice, myocardial performance index can be used to assess subclinical damage of systolic and diastolic LV function. Even when echocardiography reveals no changes in cardiac function, DCM should not be excluded and further investigations are to be performed. Since MRI and other advanced imaging techniques do not have a cost-effectiveness benefit and have not been yet validated as reliable methods for this purpose, there is novel biomarker of increased interest to identify early signs of cardiac functional alterations. In this context, miRNA might represent a valuable instrument, colligated with MMPs and PIIINP levels in order to achieve landmarks of the metabolic and functional cardiac status. As for therapeutic and prognostic values, exosomes show a promising role, but further thoroughness must be achieved for understanding their place in the convergence of various pathways.

## Figures and Tables

**Table 1:** DCM classification (adapted from Maisch et al., 2011).

Classification landmarks	Stage 1 DCM (diastolic dysfunction, hypertrophy)	Stage 2 DCM systolic dysfunction and dilatation	Stage 3 DCM systolic dysfunction, dilatation, associated HTA	Stage 4 DCM including all confounders, also CAD
Correspondence in NYHA classification	Asymptomatic	NYHA II	NYHA II-III	NYHA II–IV
Metabolic status	Impaired glucose tolerance; metabolic syndrome	Chronic hyperglycemia	Insulin resistance; DM with microangiopathic complications	DM with micro- and macroangiopathic complications
Echocardiographic features ± coronarography	Increased LV mass, diastolic dysfunction, decreased tissue velocities, normal EF	Increased LV mass and wall thickness, diastolic and systolic dysfunction (EF < 50%) mild cavity dilatation	Diastolic dysfunction and mild systolic dysfunction cavity dilatation	Moderate-severe systolic dysfunction cavity dilatation associated coronary artery disease
Other DM-related associated comorbidities			Microangiopathic complications; HTA	Macroangiopathic complications, including CAD
Serological markers to be monitored periodically regarding glycemic control, heart failure, and myocardial necrosis	NTproBNP, MMP-3, and osteopontin (according to van der Leeuw et al. 2016) Glc, lipid profile, HbA1C	MMP-3 and osteopontin (according to van der Leeuw et al. 2016) Glc, lipid profile, HbA1C, NTproBNP, BNP	MMP-3 and osteopontin (according to van der Leeuw et al. 2016) Glc, lipid profile, HbA1C, NTproBNP, BNP troponins increased in concurrent ischemia	Glc, lipid profile, HbA1C, NTproBNP, BNP troponins increased in myocardial infarction or severe heart failure

DDM: diabetes mellitus; EF: ejection fraction; Glc: glucose level;

HTA: arterial hypertension; LV: left ventricle; and MMP-3: metalloproteinase 3.

## References

[1] CDC (2011). *National Diabetes Fact Sheet: National Estimates and General Information on Diabetes and Prediabetes in the United States, 2011*.

[2] Bayeva M., Sawicki K. T., Ardehali H. (2013). Taking diabetes to heart—deregulation of myocardial lipid metabolism in diabetic cardiomyopathy. *Journal of the American Heart Association*.

[3] From A. M., Leibson C. L., Bursi F. (2006). Diabetes in heart failure: prevalence and impact on outcome in the population. *American Journal of Medicine*.

[4] Berry C., Brett M., Stevenson K., McMurray J. J. V., Norrie J. (2008). Nature and prognostic importance of abnormal glucose tolerance and diabetes in acute heart failure. *Heart*.

[5] Falcão-Pires I., Leite-Moreira A. F. (2012). Diabetic cardiomyopathy: understanding the molecular and cellular basis to progress in diagnosis and treatment. *Heart Failure Reviews*.

[6] Yildirim S. S., Akman D., Catalucci D., Turan B. (2013). Relationship between downregulation of miRNAs and increase of oxidative stress in the development of diabetic cardiac dysfunction: junctin as a target protein of miR-1. *Cell Biochemistry and Biophysics*.

[7] Loncarevic B., Trifunovic D., Soldatovic I., Vujisic-Tesic B. (2016). Silent diabetic cardiomyopathy in everyday practice: a clinical and echocardiographic study. *BMC Cardiovascular Disorders*.

[8] Maisch B., Alter P., Pankuweit S. (2011). Diabetic cardiomyopathy—fact or fiction?. *Herz*.

[9] Ernande L., Bergerot C., Rietzschel E. R. (2011). Diastolic dysfunction in patients with type 2 diabetes mellitus: is it really the first marker of diabetic cardiomyopathy?. *Journal of the American Society of Echocardiography*.

[10] Pattoneri P., Sozzi F. B., Catellani E. (2008). Myocardial involvement during the early course of type 2 diabetes mellitus: usefulness of Myocardial Performance Index. *Cardiovascular Ultrasound*.

[11] Chaudhary A. K., Aneja G. K., Shukla S., Razi S. M. (2015). Study on diastolic dysfunction in newly diagnosed type 2 diabetes mellitus and its correlation with glycosylated haemoglobin (HbA1C). *Journal of Clinical and Diagnostic Research*.

[12] Wolf P., Winhofer Y., Krssak M. (2016). Suppression of plasma free fatty acids reduces myocardial lipid content and systolic function in type 2 diabetes. *Nutrition, Metabolism and Cardiovascular Diseases*.

[13] Shah R. V., Abbasi S. A., Kwong R. Y. (2014). Role of cardiac MRI in diabetes. *Current Cardiology Reports*.

[14] van der Leeuw J., Beulens J. W., van Dieren S. (2016). Novel biomarkers to improve the prediction of cardiovascular event risk in type 2 diabetes mellitus. *Journal of the American Heart Association*.

[15] Pappachan J. M., Varughese G. I., Sriraman R. (2013). Diabetic cardiomyopathy: pathophysiology, diagnostic evaluation and management. *World Journal of Diabetes*.

[16] Furuhashi M., Fucho R., Görgün C. Z., Tuncman G., Cao H., Hotamisligil G. S. (2008). Adipocyte/macrophage fatty acid-binding proteins contribute to metabolic deterioration through actions in both macrophages and adipocytes in mice. *Journal of Clinical Investigation*.

[17] Cao H., Maeda K., Gorgun C. Z. (2006). Regulation of metabolic responses by adipocyte/macrophage fatty acid-binding proteins in leptin-deficient mice. *Diabetes*.

[18] Heyliger C. E., Scarim A. L., Eymer V. P., Skau K. A., Powell D. M. (1997). Characteristics of the myocardial PM-FABP: effect of diabetes mellitus. *Molecular and Cellular Biochemistry*.

[19] Bagheri R., Qasim A. N., Mehta N. N. (2010). Relation of plasma fatty acid binding proteins 4 and 5 with the metabolic syndrome, inflammation and coronary calcium in patients with type-2 diabetes mellitus. *American Journal of Cardiology*.

[20] Avlas O., Fallach R., Shainberg A., Porat E., Hochhauser E. (2011). Toll-like receptor 4 stimulation initiates an inflammatory response that decreases cardiomyocyte contractility. *Antioxidants and Redox Signaling*.

[21] Pal D., Dasgupta S., Kundu R. (2012). Fetuin-A acts as an endogenous ligand of TLR4 to promote lipid-induced insulin resistance. *Nature Medicine*.

[22] Jensen M. K., Bartz T. M., Mukamal K. J. (2013). Fetuin-A, type 2 diabetes, and risk of cardiovascular disease in older adults: the cardiovascular health study. *Diabetes Care*.

[23] Jialal I., Devaraj S., Bettaieb A., Haj F., Adams-Huet B. (2015). Increased adipose tissue secretion of Fetuin-A, lipopolysaccharide-binding protein and high-mobility group box protein 1 in metabolic syndrome. *Atherosclerosis*.

[24] Cohen K., Waldman M., Abraham N. G. (2017). Caloric restriction ameliorates cardiomyopathy in animal model of diabetes. *Experimental Cell Research*.

[25] Noyan H., El-Mounayri O., Isserlin R. (2015). Cardioprotective signature of short-term caloric restriction. *PLoS ONE*.

[26] León L. E., Rani S., Fernandez M., Larico M., Calligaris S. D. (2016). Subclinical detection of diabetic cardiomyopathy with MicroRNAs: challenges and perspectives. *Journal of Diabetes Research*.

[27] Tang M., Li J., Huang W. (2010). Proteasome functional insufficiency activates the calcineurin-NFAT pathway in cardiomyocytes and promotes maladaptive remodelling of stressed mouse hearts. *Cardiovascular Research*.

[28] Hu J., Klein J. D., Du J., Wang X. H. (2008). Cardiac muscle protein catabolism in diabetes mellitus: activation of the ubiquitin-proteasome system by insulin deficiency. *Endocrinology*.

[29] Li J., Ma W., Yue G. (2017). Cardiac proteasome functional insufficiency plays a pathogenic role in diabetic cardiomyopathy. *Journal of Molecular and Cellular Cardiology*.

[30] Guo R., Zhang Y., Turdi S., Ren J. (2013). Adiponectin knockout accentuates high fat diet-induced obesity and cardiac dysfunction: role of autophagy. *Biochimica et Biophysica Acta—Molecular Basis of Disease*.

[31] Bhuiyan S., Pattison J. S., Osinska H. (2013). Enhanced autophagy ameliorates cardiac proteinopathy. *Journal of Clinical Investigation*.

[32] Petrova R., Yamamoto Y., Muraki K. (2002). Advanced glycation endproduct-induced calcium handling impairment in mouse cardiac myocytes. *Journal of Molecular and Cellular Cardiology*.

[33] Jiang X., Liu W., Deng J. (2013). Polydatin protects cardiac function against burn injury by inhibiting sarcoplasmic reticulum Ca^2+^ leak by reducing oxidative modification of ryanodine receptors. *Free Radical Biology and Medicine*.

[34] Yan D., Luo X., Li Y. (2014). Effects of advanced glycation end products on calcium handling in cardiomyocytes. *Cardiology*.

[35] Sung M. M., Hamza S. M., Dyck J. R. B. (2015). Myocardial metabolism in diabetic cardiomyopathy: potential therapeutic targets. *Antioxidants and Redox Signaling*.

[36] Ng A. C. T., Delgado V., Bertini M. (2010). Myocardial steatosis and biventricular strain and strain rate imaging in patients with type 2 diabetes mellitus. *Circulation*.

[37] Towner R. A., Smith N., Saunders D. (2015). In vivo targeted molecular magnetic resonance imaging of free radicals in diabetic cardiomyopathy within mice. *Free Radical Research*.

[38] Miki T., Yuda S., Kouzu H., Miura T. (2013). Diabetic cardiomyopathy: pathophysiology and clinical features. *Heart Failure Reviews*.

[39] Faria A., Persaud S. J. (2017). Cardiac oxidative stress in diabetes: mechanisms and therapeutic potential. *Pharmacology & Therapeutics*.

[40] Yilmaz S., Canpolat U., Aydogdu S., Abboud H. E. (2015). Diabetic cardiomyopathy; summary of 41 years. *Korean Circulation Journal*.

[41] Thum T., Gross C., Fiedler J. (2008). MicroRNA-21 contributes to myocardial disease by stimulating MAP kinase signalling in fibroblasts. *Nature*.

[42] Tan Y., Ichikawa T., Li J. (2011). Diabetic downregulation of Nrf2 activity via ERK contributes to oxidative stress-induced insulin resistance in cardiac cells in vitro and in vivo. *Diabetes*.

[43] Ebisuya M., Kondoh K., Nishida E. (2005). The duration, magnitude and compartmentalization of ERK MAP kinase activity: mechanisms for providing signaling specificity. *Journal of Cell Science*.

[44] Yellon D. M., Davidson S. M. (2014). Exosomes nanoparticles involved in cardioprotection?. *Circulation Research*.

[45] Westermeier F., Riquelme J. A., Pavez M. (2016). New molecular insights of insulin in diabetic cardiomyopathy. *Frontiers in Physiology*.

[46] Garcia N. A., Moncayo-Arlandi J., Sepulveda P., Diez-Juan A. (2016). Cardiomyocyte exosomes regulate glycolytic flux in endothelium by direct transfer of GLUT transporters and glycolytic enzymes. *Cardiovascular Research*.

[47] Di Mauro V., Iafisco M., Salvarani N. (2016). Bioinspired negatively charged calcium phosphate nanocarriers for cardiac delivery of MicroRNAs. *Nanomedicine*.

[48] Rusconi F., Ceriotti P., Miragoli M. (2016). Peptidomimetic targeting of Cav*β*2 overcomes dysregulation of the L-type calcium channel density and recovers cardiac function. *Circulation*.

